# Differences in fractal patterns and characteristic periodicities between word salads and normal sentences: Interference of meaning and sound

**DOI:** 10.1371/journal.pone.0247133

**Published:** 2021-02-18

**Authors:** Jun Shimizu, Hiromi Kuwata, Kazuo Kuwata

**Affiliations:** 1 United Graduate School of Drug Discovery and Medical Information Sciences, Tokai National Higher Education and Research System, Gifu University, Gifu, Japan; 2 Dept. of Pediatric Nursing, Shiga University of Medical Science, Otsu, Japan; Nagasaki University Graduate School of Biomedical Sciences, JAPAN

## Abstract

Fractal dimensions and characteristic periodicities were evaluated in normal sentences, computer-generated word salads, and word salads from schizophrenia patients, in both Japanese and English, using the random walk patterns of vowels.　In normal sentences, the walking curves were smooth with gentle undulations, whereas computer-generated word salads were rugged with mechanical repetitions, and word salads from patients with schizophrenia were unreasonably winding with meaningless repetitive patterns or even artistic cohesion. These tendencies were similar in both languages. Fractal dimensions between normal sentences and word salads of schizophrenia were significantly different in Japanese [1.19 ± 0.09 (n = 90) and 1.15 ± 0.08 (n = 45), respectively] and English [1.20 ± 0.08 (n = 91), and 1.16 ± 0.08 (n = 42)] (p < 0.05 for both). Differences in long-range (>10) periodicities between normal sentences and word salads from schizophrenia patients were predominantly observed at 25.6 (p < 0.01) in Japanese and 10.7 (p < 0.01) in English. The differences in fractal dimension and characteristic periodicities of relatively long-range (>10) presented here are sensitive to discriminate between schizophrenia and healthy mental state, and could be implemented in social robots to assess the mental state of people in care.

## Introduction

Language is generally regarded as a one-dimensional array working in a multidimensional space where characters interact with each other, from short to long-range, resulting in the formation of particular patterns, such as words and sentences. Such patterns typically represent meaning, and include human thoughts, feelings, emotions, will, and knowledge. Words are generated by choosing an appropriate word from multiple synonyms that possess various nuances (homonymy or polysemy) [1–5], and during that selection, the acoustic or sound image of signs is referred to. The interference between the sound image and the meaning discriminates the natural language from programming language or mathematical language.

Language dysfunction is a central symptom in schizophrenia [6] and is reportedly associated with thought disorder [6–12], impaired grammatical processing [13], verbal communication disturbances [14], and disorder in the structure building framework [15]. While it was reported that the usage of total number of vocabulary in sentences represented by type token ratio (TTR) was somewhat limited to detect the symptoms in schizophrenia [16]. Recently, incoherence in speech in schizophrenia was reported and quantified compared with healthy controls [17]. Normative associations in the speech of individuals at familial high-risk for schizophrenia were reportedly decreased [18]. Hence, the density of meaning in words or sentences may be different between individuals with and without schizophrenia.

Fractal analyses to assign a fractal dimension which is a measure of complexity of sentences were also applied to extract keywords in articles [19]. Text can be understood through regularities in the spatial distribution of characters or words [20]. Previous studies have shown that the regularity of words in text could be explained as a power law relationship, and patterns of words were reported [20]. In general, text is not just a random collection of characters or words, and the density of meaning in the text varies between individuals. The density of meaning may vary from low, such as in a “word salad” generated by a computer or patient with schizophrenia, to high, such as in literature produced by a novelist without schizophrenia. The normal ordering of characters in words are also impaired in word salads produced by patients with schizophrenia. Analysis of meaningless texts and/or words needs to start with analysis at the character level rather than the word level. The characters in the text may be placed in a specific order to impart meaning. Zipf’s law [20] states that given a large sample of words used, the frequency of any word is inversely proportional to its rank in the frequency table. So word number n has a frequency proportional to 1/n. However, it has not been reported whether Zipf’s law remains unchanged with random shuffling of characters in text, as is typically seen in word salads, drastically destroying the meaning of a text [19].

Cyclic periodicity of words in sentences was analyzed using Fourier transformation that decomposes a function) (often a function of time) into its constituent frequencies, and long-range periodic cycles associated with the collective emotions were reported [19, 20]. DNA sequence also contains one-dimensional information and can be read along a time axis. We previously analyzed self-similarity in various DNA sequences using Fourier transformation [21]. We found some characteristic periodicities in DNA sequences, especially in the intron region, such as 21.3 base pair (bp) or 10.6 bp [21] which corresponds to the length of single turn free DNA double strand [22, 23], and discussed the evolution of periodicity in the DNA sequence [21].

The meaning of a text may be supported by maintaining the grammatical rules that determine where the characters, which define words, should be placed within a sentence to make short-range correlations between the sequences of characters in a sentence, although there are many redundancies due to multiple synonyms and paraphrases to maintain a similar meaning. These paraphrases may also construct long-range correlations between characters in a sentence depending on complicated conditions, such as the mental state of author. This may occur due to selected characters or words representing logical thinking at one side, but also include the emotional aspect at the other side. In logical thinking, the meaning may be independent of sound, whereas sound, such as music, may be essential to emotional thinking. It may be difficult to remove the emotional contribution to logical thinking process because we primarily connect words according to the logical meaning; nonetheless, it is difficult to randomize the sounds in texts. In normal sentences, the logical contribution is expected to be dominant; however, no quantitative or qualitative indices have been reported that represent the density of meaning in the sentence. Thus, it is essential to develop an indicator to discriminate the density of meaning in a given text to provide a very useful starting point to evaluate the human mental state, such as schizophrenia.

**Fig 1:**
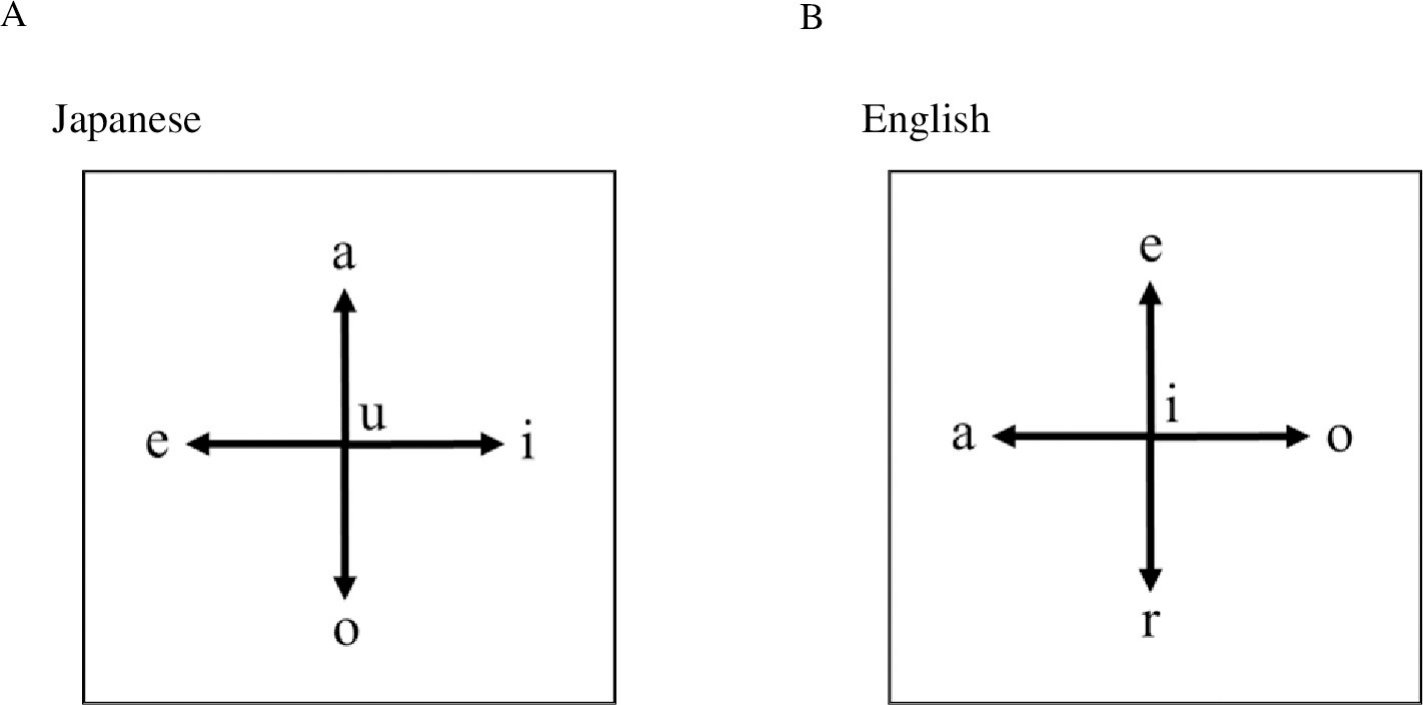
Rules of random walk. Rules of the random walk (A) Initial position of the text was always (0, 0) in the (x, y) coordinates. In Japanese, when i-th vowel was “a”, then the i-th position increased by one in the y coordinate [i.e., (x_i_, y_i_) = (x_i-1_, y_i-1_+1)]. When the i-th vowel was “i”, then (x_i_, y_i_) = (x_i-1_+1, y_i-1_). When the i-th vowel was “e”, then (x_i_, y_i_) = (x_i-1_−1, y_i-1_). When the i-th vowel was “o”, then (x_i_, y_i_) = (x_i-1_, y_i-1_−1). On the other hand, when the i-th vowel was “u”, then it remained in the same position [i.e., (x_i_, y_i_) = (x_i-1_, y_i-1_)]. (B) In English, the rule was only conventional, i.e. when i-th character was “e”, then the i-th position increased by one in the y coordinate [i.e., (x_i_, y_i_) = (x_i-1_, y_i-1_+1)]. When the i-th character was “o”, then (x_i_, y_i_) = (x_i-1_+1, y_i-1_). When the i-th character was “a”, then (x_i_, y_i_) = (x_i-1_−1, y_i-1_). When the i-th vowel was “r”, then (x_i_, y_i_) = (x_i-1_, y_i-1_−1). On the other hand, when the i-th vowel was “i”, then it remained in the same position [i.e., (x_i_, y_i_) = (x_i-1_, y_i-1_)].

The present study mainly focused on vowels in texts generated by healthy individuals (normal controls), computer-generated word salads (computer salads), and word salads from patients with schizophrenia (human salads). Here we applied the meaning independent methods, i.e. fractal and Fourier analyses. To do that we initially defined the rule of random walk as shown in Fig 1 and used the fractal concept (a mathematical object that is characterized by self-similarity) to characterize the complexity at the character level in a given text. The fractal dimension shows how the detail of a fractal pattern changes with scale, and is used as an index of complexity. The positions of a vowel within the text array form a fractal pattern with a specified dimension. We calculated the differences in fractal dimensions between three categories (normal sentences, computer-generated word salads, and word salads from patients with schizophrenia) and also examined the characteristic periodicities using Fourier transformation between three groups in both Japanese and English to characterize the internal structure embedded in the texts, and discussed the interpretation of the calculated results from the view point of a classic psychiatric theory [24].

## Methods

### Text selection in six groups

For normal conversations in Japanese (NJ), 90 texts were selected from articles found in Japanese newspapers (Yomiuri-Shinbun and Kyoto-Shinbun), blogs, and E-books. The content of the newspapers and blogs included fashion, politics, culture, sports, hobbies, medicine, and everyday life. E-books were selected from various categories, including detective, mystery, history, and romance novels. Texts were selected by shuffling the texts and selecting them for further analyses using a table of random numbers.

Ninety computer-generated word salads in Japanese (CJ) were generated using a Japanese word salad generator (https://anagram.httqs.com/). For the input, we used the same condition described for NJ, including randomization.

Word salads in Japanese (SJ) were obtained by searching the website of the Non-Profit Organization (NPO) Japan Medical Abstracts Society (Ichushi-Web) and 45 words salads were selected from published articles on psychological diseases. Selection criteria were as follows: 1) Diagnosis must be categorized as axis II in DSM 5 [25] (schizophrenia spectrum and other psychotic disorders) or included axis II. 2) Words or sentences of the patients must be unintelligible and long enough (> 128 characters) for the analyses. Summary of patients is listed in S1 Table, and examples in SJ are shown in S1 File. Obtained number of nouns to number of characters ratios for NJ, CJ and SJ were 0.091 ± 0.032, 0.083 ± 0.023 and 0.10 ± 0.017, respectively, and there were no statistically significant differences between them.

For normal conversations in English (NE), 91 texts were selected among the articles in English newspapers (The Asahi Shimbun and The Japan Time), blogs, and E-books. The content of the newspapers and blogs included fashion, politics, culture, sports, hobbies, medicine, and everyday life. E-books were selected from multiple categories, including detective, mystery, history, and romance novels. Texts were selected by shuffling all the texts and selecting those in Japanese.

Computer-generated word salads in English (CE) were generated using a word salad generator in English (http://cadrpear.tx0.org/wordsalad/salad.html). For the input, the same conditions as described above in NE were used, including randomization.

Word salads in English (SE) were obtained by searching PubMed (https://pubmed.ncbi.nlm.nih.gov/) using the keyword “word salad” and selected 42 words salads in published articles on psychological diseases. Selection criteria were as follows: 1) Diagnosis must be categorized as axis II in DSM 5 [25] (schizophrenia spectrum and other psychotic disorders) or include axis II. 2) Words or sentences of the patients must be unintelligible and long enough (> 128 characters) for the analyses. Summary of patients is listed in S2 Table, and examples in SE are shown in S1 File. Obtained number of nouns to number of characters ratios for NE, CE and SE were 0.044 ± 0.012, 0.057 ± 0.005 and 0.044 ± 0.010, respectively, and there were no statistically significant differences between them.

### Random walks in texts

The rules of the random walks in Japanese and English were defined as shown in Fig 1A and 1B, respectively, although other configurations could not be excluded. Axis rotation should not have changed properties such as the fractal dimension or periodicity. In our rule, the initial position of the text was always (0, 0) in the (x, y) coordinates. In Japanese, when i-th vowel was “a” (i.e., “a,” “ka,” “sa,” “ta,” “na,” “ha,” “ma,”“ya,” “la,” or “wa”), then the i-th position increased by one in the y coordinate [i.e., (x_i_, y_i_) = (x_i-1_, y_i-1_+1)]. When the i-th vowel was “i” (i.e., “i,” “ki,” “si,” “ti,” “ni,” “hi,” “mi,” or “ri,”), then (x_i_, y_i_) = (x_i-1_+1, y_i-1_). When the i-th vowel was “e” (i.e., “e,” “ke,” “se,” “te,” “ne,” “he,” “me,” or “re”), then (x_i_, y_i_) = (x_i-1_−1, y_i-1_). When the i-th vowel was “o” (i.e., “o,” “ko,” “so,” “to,” “no,” “ho,” “mo,” “yo,” or “ro”), then (x_i_, y_i_) = (x_i-1_, y_i-1_−1). On the other hand, when the i-th vowel was “u” (i.e., “u,” “ku,” “su,” “tu,” “nu,” “hu,” “mu,” “yu,” or “ru”), then it remained in the same position [i.e., (x_i_, y_i_) = (x_i-1_, y_i-1_)]. We ignored punctuation characters and space for analysis. All Kanji (Chinese characters) were converted to Kana (Japanese syllabaries), and then above rules were applied.

Since vowels in English are more complicated than those of Japanese, just for comparison, we selected five characters, i.e. when i-th character was “e”, then the i-th position increased by one in the y coordinate [i.e., (x_i_, y_i_) = (x_i-1_, y_i-1_+1)]. When the i-th character was “o”, then (x_i_, y_i_) = (x_i-1_+1, y_i-1_). When the i-th charcater was “a”, then (x_i_, y_i_) = (x_i-1_−1, y_i-1_). When the i-th vowel was “r”, then (x_i_, y_i_) = (x_i-1_, y_i-1_−1). On the other hand, when the i-th character was “i”, then it remained in the same position [i.e., (x_i_, y_i_) = (x_i-1_, y_i-1_)]. We ignored punctuation characters and spaces for analysis. Also the analyses were case-insensitive. Although there are many ways for selection of characters in English, and the rule described above is just conventional, but generality is not lost in view of fractal analyses and Fourier transformation.

### Determination of fractal dimensions

Text is a specific arrangement of characters in a one-dimensional array that carries meaning. Random rearrangement of the characters across the text significantly reduces its meaning; hence, the ordering of the characters is important to the meaning. In other words, the meaning shows regularity in a text, which may manifest itself in a pattern of occurrence of each character in the text array. If we consider the text array as a one-dimensional space, the spatial pattern of occurrence of any character will form a fractal set or simply a fractal. We can assign a fractal dimension to any character in a given text using the practical method of box counting using software, such as the box count routine in MatLab (Mathworks) or Fractal Analysis System by Hiroyuki Sasaki at National Agriculture and Food Research Organization. Using this method, the obtained fractal dimension of random walk was between 1 and 2. IBM SPSS Statistics Base (version 26) was used for statistical analysis (one-way ANOVA).

### Fourier analyses

Microsoft Excel 2016 functions were used to perform Fast Fourier transform analyses and power spectra were obtained as a function of periodicity. Baseline biases were removed by calculating the difference in periodicities between each pair of groups and statistically evaluating the significance of each periodicity. IBM SPSS Statistical Base (version 26) was used for statistical analysis (one-way ANOVA).

## Results

### Universal properties of texts

**Fig 2:**
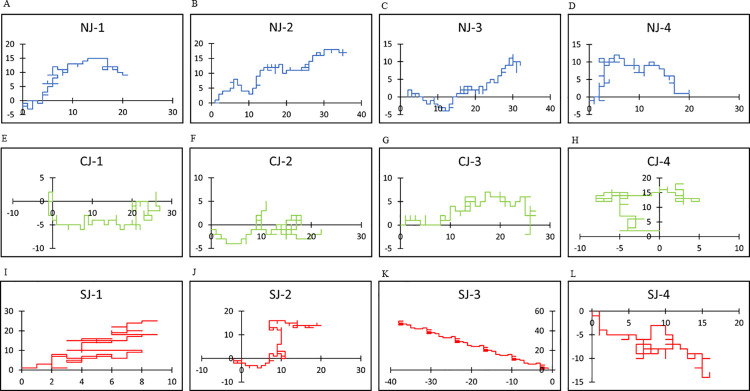
Representative plots of random walks in Japanese. Horizontal axis represents (x_i_) and vertical axis represents (y_i_) according to the rule in Fig 1A. (A-D): Representative random walks in Japanese for normal sentences (NJ) are plotted in blue, (E-H): Those for computer generated word salad (CJ) are plotted in green, (I-L): Those for word salads are plotted in red.

**Fig 3:**
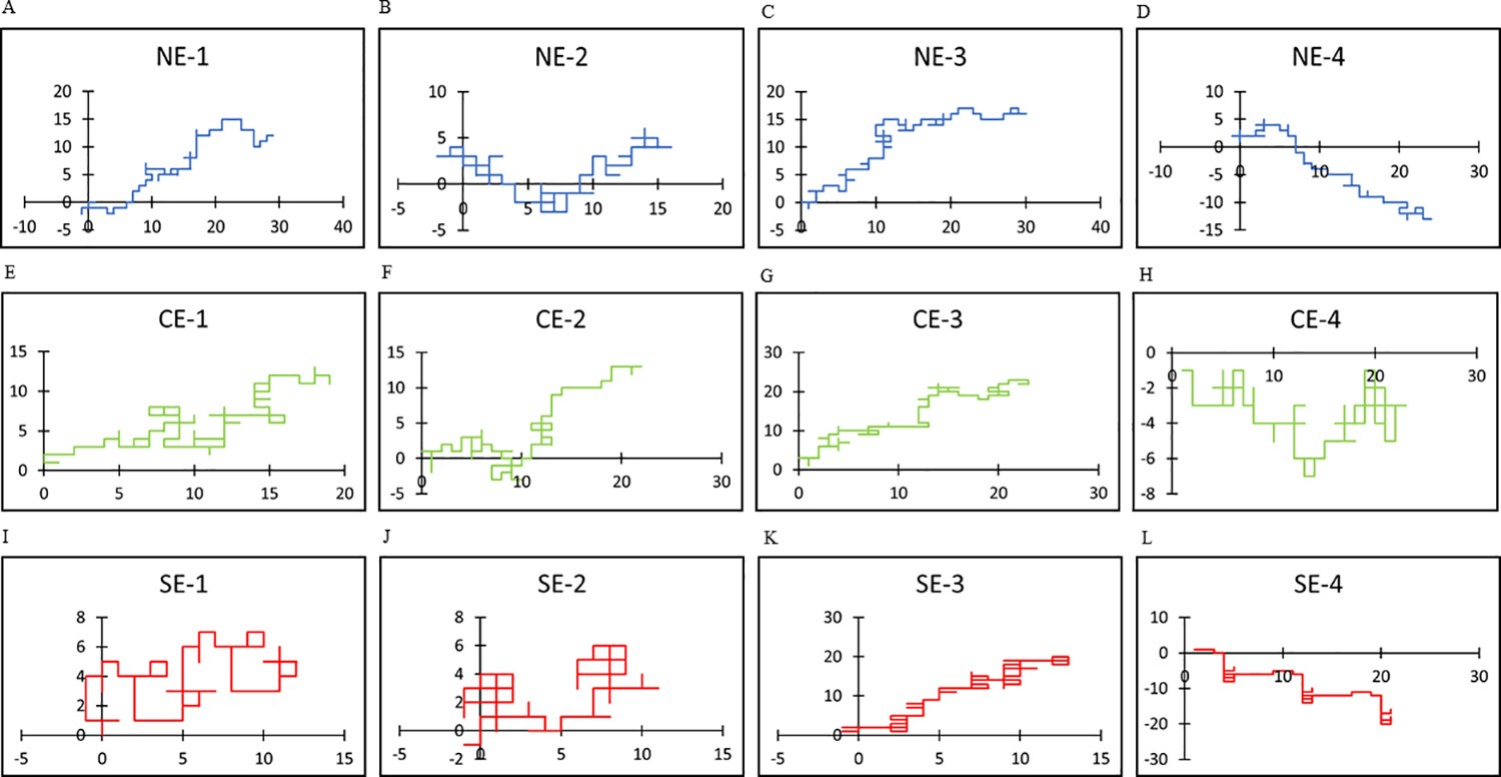
Representative plots of random walks in English. Horizontal axis represents (x_i_) and vertical axis represents (y_i_) according to the rule in Fig 1B. (A-D): Representative random walks in English for normal sentences (NE) are plotted in blue, (E-H): Those for computer generated word salad (CE) are plotted in green, (I-L): Those for word salads (SE) are plotted in red.

All random walk patterns for the utilized texts are shown in S1 Fig. In Japanese texts, the use of “i” was dominant compared with the use of “e,” and the use of “a” was slightly dominant compared with that of “o”. Similar tendencies were also observed in English, in that the use of “e” or “o” was predominant compared with that of “r” or “a”, respectively. Several representative curves were plotted to show the details of the random walks (Figs 2 and 3). Random walk patterns were markedly different between the three groups NJ and NE, CJ and CE, and SJ and SE). In normal conversations (NJ and NE), the curves were smooth with gentle undulations, whereas in computer-generated word salads (CJ and CE), they were rugged with mechanical repetitions. Word salads generated from patients with schizophrenia (SJ and SE) were unreasonably winding with a meaningless repetitive pattern or even artistic cohesion. These tendencies were similar in both languages.

### Determination of fractal dimensions

In box counting, the space is divided into boxes and each box that contains a component of the fractal set is called a filled box. The fractal law is a power law relationship between the number of filled boxes and the box size. To calculate the fractal dimension of a character using box-counting method, the text array is divided into boxes of size *s*, and each *s* consecutive character is placed in a box. The number of such boxes is *N*_*s*_ = *N*/*s*, where *N* is the length of the text. If the considered character appears in one of the boxes, that box is a filled box, and N_b_ (s) stands for the number of filled boxes. A power law relationship exists between the number of filled boxes and the box size, s, as follows: N_b_(s) ∝ s^-D^, where D is the fractal dimension of the character. The fractal dimension is obtained by measuring the slope of log–log plot of N_b_ (s) versus s.

**Fig 4:**
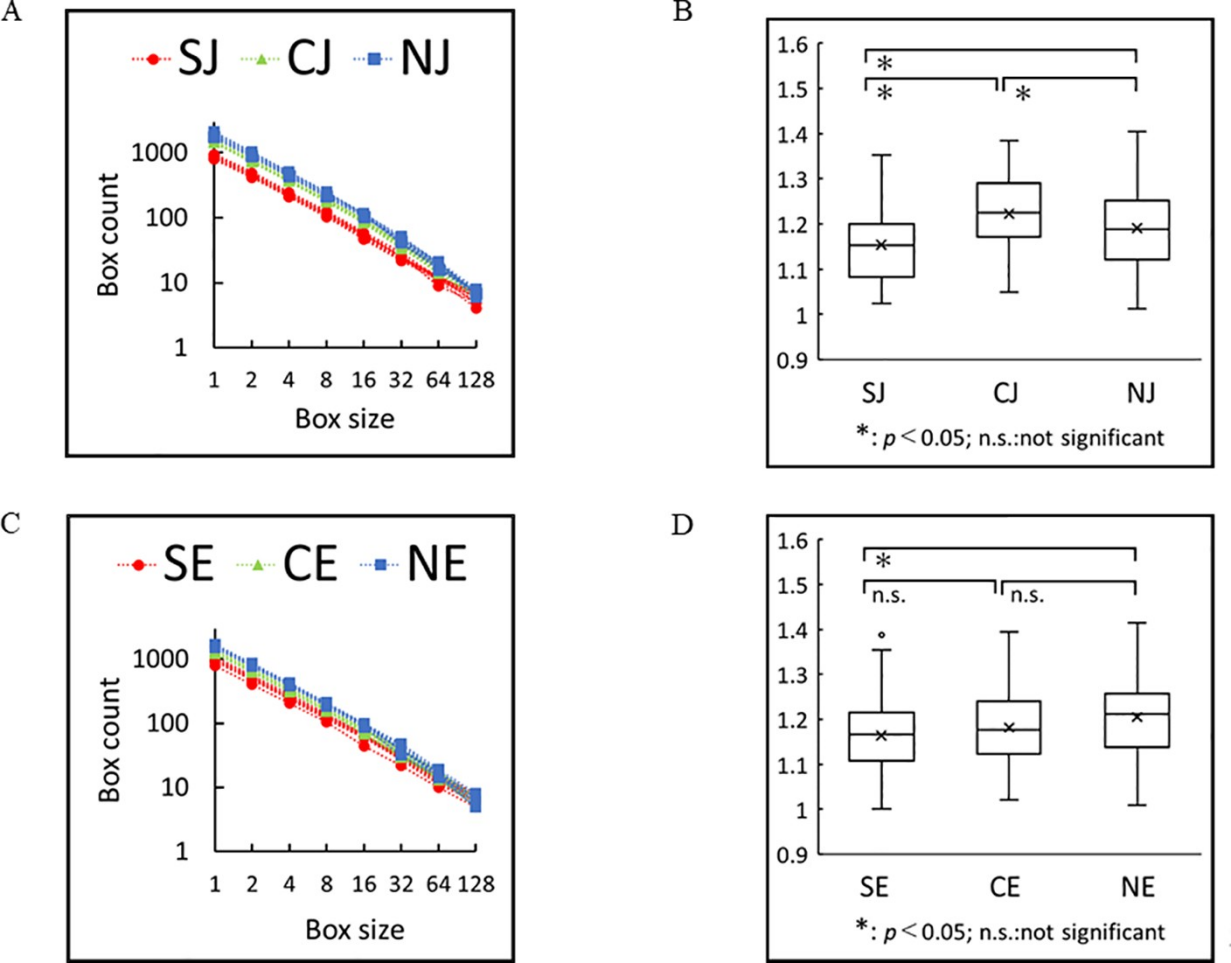
Fractal dimension by box-counting method. (A) Log-log plot of the box size versus box count in Japanese. Normal sentences (NJ), computer generated word salads (CJ) and word salads of patients (SJ) were shown in blue, green and red, respectively. It is worth noting that the box size is an integer number, and in practice, we expect to see the power law behavior for the large box sizes. (B) Differences between all three groups were significant. (P < 0.05) (C) Log-log plot of the box size versus box count in English. Normal sentences (NE), computer generated word salads (CE) and word salads of patients (SE) were shown in blue, green and red, respectively. (D) Differences between (SE) and (NE) were significant. (P < 0.05).

The fractal dimension was calculated for the six groups (Fig 4). The fractal dimensions for these random walks showed clear distinctions: 1.19 ± 0.09 (n = 90), 1.22 ± 0.08(n = 90), 1.15 ± 0.08 (n = 45), 1.20 ± 0.08 (n = 91), 1.18 ± 0.08 (n = 90), and 1.16 ± 0.08 (n = 42) for NJ, CJ, SJ, NE, CE, and SE, respectively. The fractal dimensions of NJ, CJ, and SJ were significantly different (p < 0.05; Fig 4B and 4D), and those for SE and SE were significantly different (p < 0.05). The fractal dimensions between normal sentences and word salads from schizophrenia patients were significantly different (p < 0.05) in both languages: 1.19 ± 0.09 (n = 90) and 1.15 ± 0.08 (n = 45), respectively, in Japanese, and 1.20 ± 0.08 (n = 91) and 1.16 ± 0.08 (n = 42), respectively, in English.

### Characteristic periodicities

**Fig 5:**
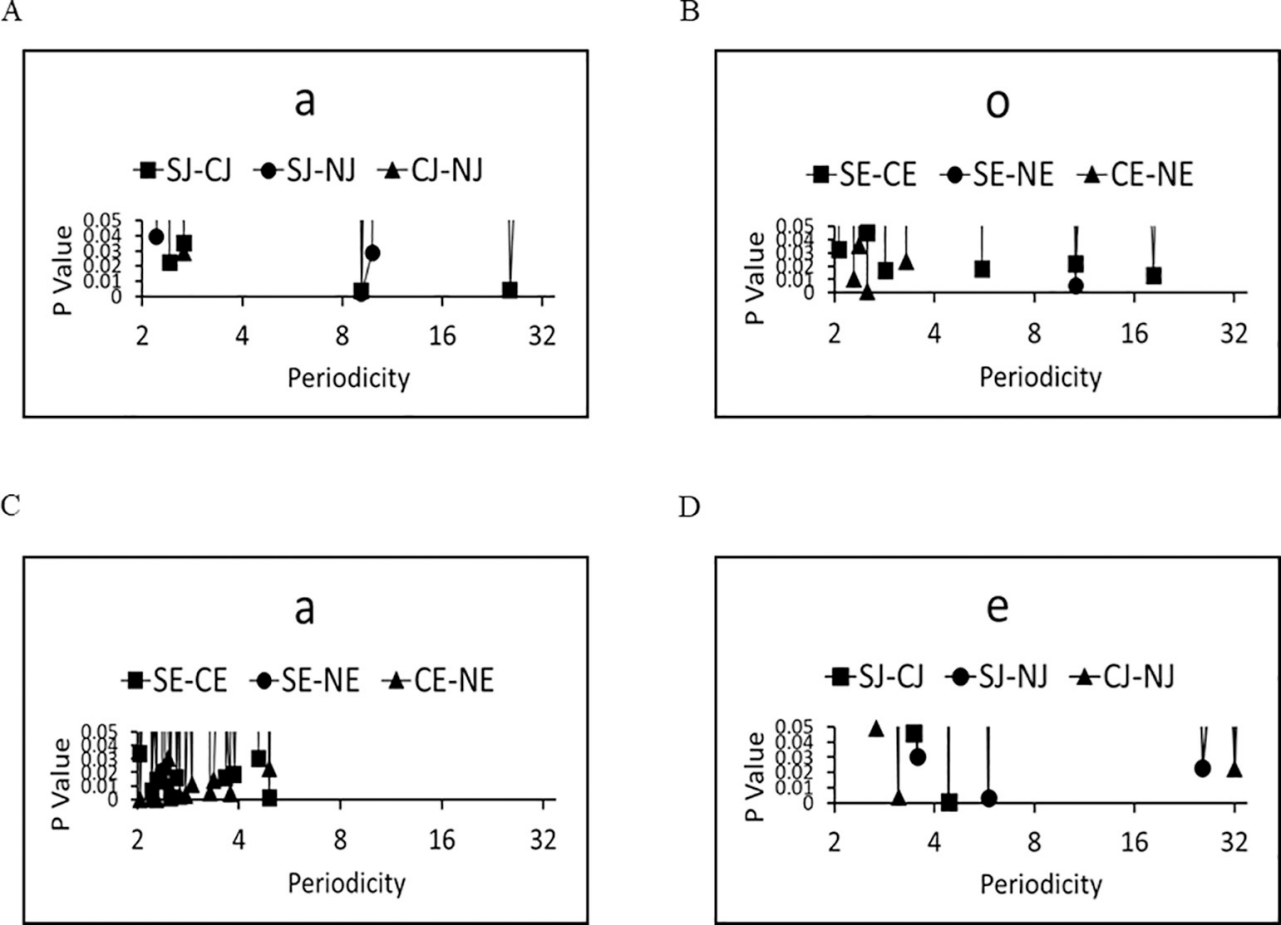
Statistically significant (characteristic) periodicities in each two groups. (A) In Japanese ‘a’, significant difference between (SJ) & (NJ), and (CJ) & (SJ) were observed at periodicities of 9.14, and also for (CJ) & (SJ) at 25.6. (B). Those in Japanese ‘a’. Significant difference between (SJ) & (NJ), and (CJ) & (SJ) were observed at periodicities of 9.14, and also for (CJ) & (SJ) at 25.6. (B) In English ‘o’, significant difference between (SE) & (NE) were observed at periodicities of 10.67. (C) In English ‘a’, significant difference between were observed at short-range periodicities (See Table 1). (D) In Japanese ‘e’, significant difference between were observed at short-range periodicities (See Table 1).

The characteristic periodicities were calculated for the six groups. The results of the whole Fourier analysis are shown in S2–S4 Figs. Characteristic periodicities with statistical significance (p < 0.01) are shown in Table 1, and the representative plots of the p-value as a function of periodicities are shown in Fig 5. Relatively long-range periodicities (>9) were observed between NJ and SJ, NE and SE, and CJ and SJ (Fig 5A and 5B and Table 1). On the other hand, relatively short-range periodicities (<4) were frequently observed when computer-generated word salads were included, such as NJ and CJ, and NE and CE (Fig 5C and 5D and Table 1). These results suggest that short-range periodicities were mainly embedded in the computer-generated word salads, whereas long-range periodicities were observed in the differences between normal sentences and word salads, suggesting that the interference between meaning and sound may occur in the long-range periodicity regions.

**Table 1:** Characteristic periodicities (p < 0.01).

Group 1	Group 2	A or a	I or e	U or i	E or o	O or r
NJ	SJ	9.14	–	–	5.82	–
NJ	CJ	–	–	2.91	3.12	2.33
						8.00
CJ	SJ	9.14	–	–	4.41	2.21
		25.6				
NE	SE	–	–	–	10.67	–
NE	CE	2.03	–	–	2.51	–
		2.21				
		2.29				
		2.51				
		2.67				
		2.78				
		3.28				
CE	SE	2.21	–	–	–	6.74
		2.51				
		4.92				

CE, computed-generated English word salads; CJ, computed-generated Japanese word salads; NE, normal English conversation; NJ, normal Japanese conversation; SE, English word salads from patients with schizophrenia; SJ, Japanese word salads from patients with schizophrenia.

## Discussion

Various meaning models, such as the latent semantic analyses [8, 17, 26] was applied to schizophrenia to analyze the incoherence of speech. They used 37,651 text samples with 92,408 unique words and matrices of 300 dimensions to perform SVD analyses. The basic question is whether it is possible to differentiate the coherence and the incoherence of speech in terms of meaning by the usage count. Rare usage or rare combination of words do not necessarily mean the incoherence in terms of meaning. We should remember that the novel scientific findings were explained only by the novel combinations of ever-existing words. Coherence of meaning in words is independent of the usage count, and is connected to cognitive function of the brain.

Computational linguistic analysis on verbal fluency has been conducted using an automated tool, CoVec [27] to detect the speech disorganization, such as derailment and tangentiality in schizophrenia. In this analysis, coherence was defined as the average similarity (norm) of each word to other words. Statistical tests (such as ANOVA) on the calculated norm was performed for pairwise comparison between control subjects and patients. However, the low similarity defined above does not necessarily mean any disorganization or abnormality in speech. Rare combinations of words with clear logical connection does not mean derailment nor tangentiality. Logical connections of words may not allow such usage count analyses, because completely new word connections, novel concepts or creative works must originate from a zero matrix element. Furthermore, more important linguistic aspects, such as acoustic characteristics [28] are still missing, and it is essentially questionable whether they could be further characterized using natural language processing (NLP) and machine learning (ML) techniques [29].

Here we employed fractal analysis and Fourier transformation methods for analyzing word salads, which are essentially independent of above meaning models. Differences in fractal dimension and characteristic periodicities between normal sentences and word salads may represent interference between meaning and sound in languages. We predominantly observed high power at relatively long-range periodicity around 6 and 9 in normal sentences in Japanese (S2A, S2K, and S2P Fig). These differences suggest that the vowel sound around the periodicity of 6 and 9 may affect the meaning. Although the working mechanism remains unclear, these characteristic periodicities are essential for understanding sentences or transmitting the exact meaning.

Several approaches have been reported regarding brain rhythmicity [30] or category fluency [31] and genes, network connectivity using a computational model [32], symptomatology and white matter tracts [33], and relationship with antipsychotic medication [34]. Although the reading mechanism of DNA sequence is well-understood biologically, the cognitive mechanism of language remains unclear. Thus, we propose a hypothetical theory based on the classic theory of psychiatry, which may explain our findings.

**Fig 6:**
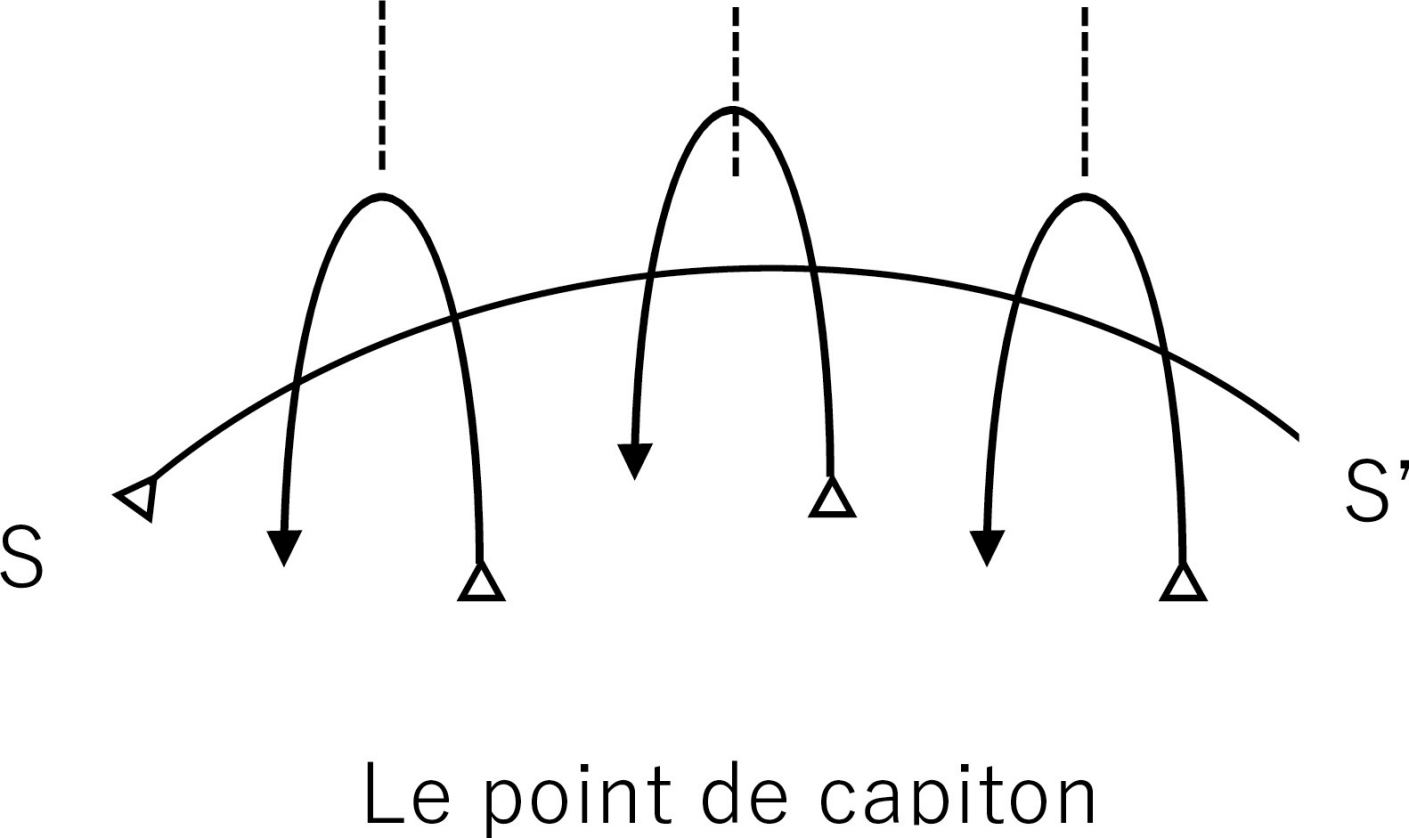
Illustration of ‘le point de capiton’. The subject sews two pieces of cloth (S and S’) together. S represent ‘signifier’ which refers to desire that leads the subject to express the sign, and S’ represent ‘signified’ which results in the signification during speech or writing. In general, S and S’ are not connected each other without ‘le point de capiton’ at appropriate intervals, which generates word salad.

In 1960, the psychiatrist, Jacques Lacan, described the notion of “le point de capiton” [24], at which in individual sews two pieces of cloth together. One cloth is referred to the “signifier”, represented by curve S in Fig 6, which denotes the desire that leads the individual to express the sign, and the other is referred to as the “signified”, represented by curve S’, which results in the signification during speech or writing [35]. Sassure proposed that the signified predominates over the signifier because the language is used to transfer the exact meaning [35], whereas Lacan argued that the signifier predominates at the level of unconsciousness and determines the logic by which symptoms are organized [24]. This indicates that the signifier and signified do not necessarily coincide. In fact, incoherence in speech has been reported in schizophrenia and quantified compared with that of healthy controls [17], and normative associations in the speech of individuals at familial high-risk of schizophrenia were reportedly decreased [18].

The present study found that the fractal dimension for normal sentences was significantly higher than that of word salads in both Japanese and English (Fig 4). A fractal dimension of 1.0 indicates a simple repetition of short segments, suggesting meaningless sentences. Hence, a fractal dimension >1.0 may correlate with the complexity of sentences i.e., the higher density of meaning. Thus density of the meaning in normal sentences are higher than those in word salads of schizophrenia both in Japanese and English. We also found that the characteristic periodicities in vowels in words salads were longer range than those of normal sentences (S2 and S3 Figs, Fig 5).

These findings suggest that the “le point de capiton” of words salads are sparser than those of normal sentences (Fig 6). If “le point de capiton” becomes sparse enough, it would lead to loosening of the association between S and S’, which corresponds to the typical symptoms of schizophrenia generating word salads. In other words, at “le point de capiton,” deeply impressive words with an emotional sound would appear to have imperturbable meaning, and the meaning of words between the “le point de capiton” would emerge spontaneously with clear meaning. “Le point de capiton” would be repeated at a long-range periodicity and the meaning of whole sentences would become deterministic.

Regardless of the detailed mechanism, the analyses used in the present study could be used to elucidate mental state, such as schizophrenia, and could also be implemented in future in social robots to assess the mental state of a person in care.

## Conclusion

The fractal dimension and Fourier analyses of human languages focusing on vowels presented in the present study useful for elucidating differences in normal sentences and various word salads. These methods are essentially independent of the meaning of the written or spoken word, and thus the relevant algorithms presented in the present study could be easily implemented in social robots to assess the mental state of a person in care. In future, we aim to construct a social robot that can communicate with people to determine their cognitive state and offer appropriate advice.

## Supporting information

S1 File (DOCX)

S1 Fig Random walks of all the data. (A) Random walks in Japanese analyzed for (A) normal sentences (NJ) are plotted in blue. (B) Random walks for computer-generated word salads (CJ) are plotted in green. (C) Random walks for word salads of schizophrenia patients (SJ) plotted in red. Random walks in English analyzed for (D) normal sentences (NE) are plotted in blue. (E) Random walks for computer generated word salad (CE) are plotted in green. (F) Random walks for word salads of schizophrenia patients (SE) are plotted in red.
(PPTX)

S2 Fig Power as a function of periodicity for random walk in Japanese. Power as a function of periodicity for random walk of all the data in Japanese: NJ (A–E), CJ (F–J), and SJ (K–O). (P–T) Mean and standard deviation of power at each integer periodicity are shown for NJ (blue), CJ (green), and SJ (red).
(PPTX)

S3 Fig Power as a function of periodicity for random walk in English. Power as a function of periodicity for random walk of all the data in English: NE (A–E), CE (F–J), and SE (K–O). (P–T) Mean and standard deviation of power at each integer periodicity are shown for NE (blue), CE (green), and SE (red).
(PPTX)

S4 Fig Calculation of the statistically significant (characteristic) periodicities in each pair of groups. (A–E) *P*-values for the difference between SJ and CJ, SJ and NJ, and CJ and NJ are plotted as a function of periodicity. (F–J) *P*-values for the difference between SE and CE, SE and NE, and CE and NE are plotted as a function of periodicity.
(PPTX)

S1 Table Summary of patients information in SJ. (DOCX)

S2 Table Summary of patients in formation in SE. (DOCX)
