# Supplementary material for: Differences in fractal patterns and characteristic periodicities between word salads and normal sentences: Interference of meaning and sound
Source: PLoS One. 2021 Feb 18;16(2):e0247133. doi: 10.1371/journal.pone.0247133 (PMC7891721; doi:10.1371/journal.pone.0247133)
Supplement: S1 File — (DOCX) [file pone.0247133.s001.docx]

Examples in SJ

1. D (fractal dimension) = 1.18

dokuto,ixtutemoitibansaki,sentikotika,ikurakakosinoawarukaxtutandesune.watasihasuguderarenakaxtutandesune.dakaradousimonakaxtutandesune.soredemoosanzyoubugasuguhayaku,sakiniixtutakedo,dousiyoumonai.beso,mixtukadakewakaranakaxtuta.kotobaga,gomasiteirundesu.hokanokotosiraba,wakaxtutekitakanzi,sorededame.tebira,sako,peku,usagin,semen,tyoutin,tyoutou.dokutoixtutemo,itibansaki,sentikotika,ikurakakosinoawarukaxtutandesune.watasihasuguderarenakaxtutandesune.

2. D = 1.06

hamigakiwositematatigautoixtuta.soide,matamatigaeta.otyatotigautowakaxtuta.mata,tigautowakaxtuta.soide,mitigoketowakaxtuta.misukoubetotigautowakaxtuta.tinbagomegadekita.gingamidetukuxtuta.okazudeoisikaxtuta.tinbanomonpeni,dekihinnogadekita.kyouha,huronihairimasita.sirayukihimegadekimasita.sensei,mitehosii.osikkoniixtutekimasu.

3. D = 1.05

niwatori,kimonuitesimaxtutamono,syoutikutatetekadogotoni,dakarazisinarunorassya,ore,sonnakotokataxtutenaikara,kutikatarenakaxtutakarasenmaimokutinitumetekunai.yowaikara,tuyokunarukotorassya,samenotamagodakaraikiteitai.niwatori,kimonuitesimaxtutamono,syoutikutatetekadogotoni,dakarazisinarunorassya,ore,sonnakotokataxtutenaikara,kutikatarenakaxtutakarasenmaimokutinitumetekunai.

4. D = 1.08

aa,tottootottoo,kotukoro,kaxtutara,kotuterako,kaxtutara,koxtuterakoxtuterako,utinoutinokoxtutekoxtutera,kokkorako,kappadekade.kappadeka.yuxtutedeka.degandereka.degandereka.arasoudaxtutakane.kattereka.kanekatekanekatekanekateka.

5. D = 1.09

ieniitaonnanohitohahahaoyasokkurinonisemonoda.ienioiteaxtutasofa-hadami-da.watasinosenakanihatamoitugaimasu.areirai,toukyougyakuryuuhouteisikidesu.indhianhahadagaakai.watasimohadagaakai.dakarawatasihaindhianda.kansensyahaeizukensagayouseitonaru.zibunmoeizukensagayouseidearu.dakarazibunhakansensyada.

Examples in NJ

1. D (fractal dimension) =1.35

kosodatewositeyukuuedeyaharikurumahanoretahougaiikana,,toomoinagaramodendouzitensyadenantokanorikixtuteimasitagamusukomosigatudesyougakkounyuugakunanodetotuzennotaityouhuryoudeomukaenadotaihendana,,naraigotohenosougeimotaihendana,,tokangaesakunenmatukarakyousyuuzyohekayoxtuteimasitakosodateyaosigotonoaimawonuxtutekayounohahontouniha-dodesitagaganbaxtutakaigaarisenzitubuzinimenkyosyouwoitadakimasitadousitemosukezyu-rugatorenaitokihadonitimotakuzisisetuwotukainantokamusukononyuugakumaenimokuhyoutasseidekimasitatuginomokuhyouhamusukonoyoutiennosotuensikinisyaonkai.

2. D = 1.40

youtiende,usagigoyawosouzisiteitara,tomodatinokutuganuretesimaimasita.sonotoki,rizityousenseiga,「yakiimogadekitakara,hatakenioide」toiimasita.tomodatiha,hadasideikanakerebanaranainode,kawaisoudana,toomoimasita.sousitara,senseiga,kakitorinohappade,kutuwotukuxtutekuremasita.kakkoyokute「bokumohosiina」toomoimasita.senseinomanewosite,zibundetukuxtutara,zyouzunidekimasita.hokanimo,hosiihitogatakusanitanode,tukurikatawoosieteagemasita.minnadetukuxtutanode,kyousituniha,kututasannoyouni,ippaikutuganarabimasita.nentyuusanni「iinaxa」tteiwarete,nentyounobokutatiha,yapparisugoidesyo,toomoimasita.

3. D = 1.40

aitewotatetekyoukansurukotoga,kotowoaradatenaisubenohitotudatokanzirudekigotogaaxtuta.kousatenwousetusitatoki,hodoukarawakaizyoseikeisatukangadetekite,mousiwakenasasouni「sumimasen.hudankonohenhatoorarenaidesuka.kokohausetukinsinandesu」toixtuta.atya-,yaxtutesimaxtuta.「konomitiwotooranakereba」「kyounoyouzigabetunohidattara」nado,「tara」「reba」bakarigaomoiukanda.saraniha,「nazezibundakega」totasyawouramukimotimodetekita.sikasi,waruinoha,miotositazibunnihokanaranai.huto,anozyoseikeisatukannokosinohikusa,mousiwakenasasounayousuwoomoidasita.kitto,kotowoaratatenaisubenanodarou.

4. D = 1.39

watasiha,koukou3nenseidezyukennamadesu.watasihatakusannokabenibutukarimasita.itibannayandakotoha,zibungadoryokusiteirutoomoxtuteitemosikennotensuuganobinakaxtutakotodesu.mosinokextukagakaettekurutabi,tensuuhasagarubakaride,hyoukamokawarazukasagarukadesita.kakomonwotoitemo,seitourituganakanakaagarazu,aserisikaarimasendesita.mawarinokotatihasiboukouhenogoukakugadondonkimaxtuteikutame,saraniaserigaookikunaxtutatoki,yuuzinga「issyoniganbarou」toixtutekuretetakotogauresikaxtutadesu.

5.

D = 1.40

eyssannoressunru-muwookarisitesyamisennozisyurenwositekitayoimamadeyaxtutekitapianomoSAXmosoudesitaga,,gakkinoensouharensyuusuruzikannihireisitezyoutatusurumonodesu.zenkainoressundesenbonzakuranoensounosubetewoosowarimasitaikkaimenikaimenoressuntohikakusurutozenkaihasoutoukoinaiyoudaxtutanode,wasurenaiutinioboenaitonarimasen.zitakudemozisyurenhasitekitandakedo,ressunru-mudezisyurenwositahougasyuutyuuryokugamasukigasurueyssanhaheyagatakusanarunodezisyurenwositaitokihahontounitasukarukyokunozisyurenwohazimetemiteomounoha,

Examples in SE

1. D (fractal dimension) = 1.12

Um-hm. Oh, hey, well, I, I, oh, I really enjoyed some communities I tried it, and the next day when I'd be going out, you know, urn, I took control like, uh, I put, um, bleach on my hair in, in California. My roommate was from Chicago and she was going to the junior college. And we lived in the Y.W.C.A. so she wanted to put it, um, peroxide on my hair, and she did, and I got up and looked at the mirror and tears came to my n eyes. Now do you understand, I was fully aware of what was going on but why couldn't I, why, why the tears? I can't understand that, can you? "Well, not very much I mean, what if I were dead? It's funeral age. Well, I, um? Now I had my toenails, uh, operated on. They're, uh, um, got infected and I wasn't able to do it but they wouldn't let me at my tools.

2. D = 1.20

They're destroying too many cattle and oil just to make soap. If we need soap when you can jump into a pool of water, and then when you go to buy your gasoline, m-my folks always thought they should, get pop but the best thing to get, is motor oil, and, money. May-may as well go there and, trade in some, pop caps and, uh, tires, and tractors to group, car garages, so they can pull cars away from wrecks, is what I believed in. So I didn't go there to get no more pop when my folks said it. I just went there to get a ice-cream cone, and some pop, in cans, or we can go over there to get a cigarette. And it was the largest thing you do to-to get cigarettes cause then you could trade off, what you owned, and go for something new, it w-it

was sentimental, and that's the only thing I needed was something sentimental, and there wasn't anything else more sentimental than that, except for knick-knacks and most knick-knacks, these cost 30 or 40 dollars to get, a good billfold, or a little stand to put on your desk.

3. D = 1.10

Kings sense jester realize tongues poetry. Words hard journal describe impossible. Religious hair coma machine idea hate. Interesting hospital interpreting description extreme encounter. Junction one distant fragile mine. Death wretched addled else finally chant mind sea backgrounds. Obey space cat disjointed languages swearing admit stranger bit dressing. Picture cake chocolate rambling UFO solar here fuse barking. Online signature basic color sleep ideas class. Dog bike cat sat sit down under sleep jump. Now later red cat boat ship house girl. The Muppet Show's music often contains words strung together that do not make sense.

4. D = 1.18

“The Happy Noodle Boy comics by Jhonen Vasquez. Hubba Hubba Zoot Zoot, a song by Carumba. Worm Quartet, a comedy music act. Drinking Out of Cups, spoken word by Dan Deacon. Hatsune Miku's song Francium. Popular website LOLCats. Rutland Weekend Television's sketch "Gibberish" by Eric Idle. Monty Python's Flying Circus. Some of the comics of Calvin And Hobbe's. A stand-up act by now deceased George Carlin included Word Salads such as "Niz fluk bwarney quando floo!" The play Dogg's Hamlet by Tom Stoppard.　The Happy Noodle Boy comics by Jhonen Vasquez. Hubba Hubba Zoot Zoot, a song by Carumba. Worm Quartet, a comedy music act. Drinking Out of Cups, spoken word by Dan Deacon. Hatsune Miku's song Francium. Popular website LOL Cats. Rutland Weekend Television's sketch "Gibberish" by Eric Idle. (D=1.18)

5. D = 1.01

I had also been seeing like demons, demon kind of objects taking my family away and stuff like that after my granda passed away. Because me and my granda were very, very close and that’s whenever the depression got even worse.”“…we were driving through LOCAL TOWN and I thought there was someone sitting on a bench and there’s eh, I’m pretty sure there is someone sitting on a bench, and then I turned and looked again and nothing was there. And then I drove up the road another wee bit and there’s another bench and the exact same thing was sitting there, and it wasnae there anymore and then I says to my ma, I was just like for fuck sake and she goes what, and she says, I says I’m seeing these visions again and she says it’s just your mind playing tricks on you, but I see them nearly every day, I see shadows nearly every day whenever I’m just walking about”

Examples in NE

1. D (fractal dimension) = 1.37

"Out with the old, in with the new. Breakups, #10yearchallenges, runway reports... There's always a reason to switch up your hair color, but one of the most appropriate times would be the start of a new year. Which is why we asked these top colorists to predict the trends that will be big in 2020. From Twilighting and natural roots to the Tumeric Latte replacement of the Pumpkin Spice Latte, there's a big color mood here for everyone. Plus a few tips and products meant to maintain your newly dyed status well into spring!2020 Hair Color Trends:1. Strategic Blonde Celebrity colorist Justin Anderson works on some major manes. Along the lines of Jennifer Aniston, Gwyneth Paltrow, Kristin Cavallari, and Margot Robbie. NBD. So obviously he's partial to perfecting blondes, but he predicts we're going to be seeing more subtle highlights this year. ""Just enough to add dimension, but keep the overall look very natural-looking,"" he says. Maintenance for this look is also pretty easy, as you can go longer in-between salon appointments with Anderson's dp HUE x Kristin Cavallari Blonding Brush, $28, that allows you to paint highlights wherever you want them.

2. D = 1.32

"Thanks for all your comments. It’s encouraging to see that at least people are starting to talk about the issue. As you know, Westerners have trouble understanding Japanese ideas about both the role of teachers and the problem of work/life balance, and this story combines the two, so it’s not surprising that we have different ideas about it. The reality of being a teacher in Japan is that it means you have almost no time to spend with your own kids because you are so busy looking after other people’s. Mind you, I suppose the same thing is true in a lot of other jobs. The problem is that many teachers have to choose between having a career and having a family, and that cannot be a good thing. In particular, there is a very high turnover of elementary school teachers these days. Many of our female students tell us that they want to teach until they are about 30, and then they want to quit so that they can start a family of their own. Surely it would be better to create a work environment where they could do both."

3. D = 1.33

"This is a guest post written by Cynthia Lopez from Place Pass. Living in a hostel can either be a wonderful experience... or hell on earth! Most of us who have frequented hostels have a horror story, or at least have heard one from someone else. The thing is, hostels really shouldn't be an unpleasant place to stay (they should be an awesome and cheap place to stay). There are certain things you can do to take hostel life from blah to brilliant. We've narrowed down 8 helpful tips to ensure that your next hostel stay is memorable for the right reasons. Keep reading to learn our expert tips for living like a king or queen in a hostel! Hostel Life: How to Live Like a King or Queen#1 - Treat Yourself to a Private Room One of the best ways to live like royalty in a hostel is to treat yourself to a night or two (or even longer!) in a private room in your hostel of choice. A private room in a hostel gives you the privacy and some perks of a hotel room, without the hefty price tag.

4. D = 1.33

"Ah, tulip season in the Netherlands. For a brief period of time, from the end of March to the beginning of May, the fields of the Dutch countryside undergo a transformation. Green (or gray, depending on the weather) fields give way to vivid stripes of flowers, carpeting the landscape in rainbows of color. A magical sight to behold, to say the least.… which, of course, means that everybody and their momma and their omaatjes will be trying to get in on that flowery action, looking to snap some supersaturated superselfies. Keukenhof is the premier destination for those looking to bask in the glories of tulips and other highly-contrived Dutchy flower creations. As a result, the park turns into a certifiable madhouse of people the second the sun shines during tulip season. Add the fact that it’s expensive for travelers on a budget—Keukenhof tickets are €17,50 per person when purchased online—and you can understand Keukenhof isn’t for everyone. So, how to see tulips in the Netherlands without suffering the crowds?

5. D = 1.41

"Last week, my friend and professional food stylist Meg, of Ain't Too Proud to Meg, stopped by the office to show us how to make the movie candy board of my dreams. And, although I'm currently swearing off candy during the workday, I'll absolutely be diving into this come Oscars night (still figuring out this year's dress code after last year's ""sweats and lipstick""). If you're looking for a way to elevate your Oscars viewing party or add some sugar to a date night in, look no further: 1.Gather your favorite candies. First, you want to start by gathering all of your favorite candy. Naturally, this was where my expertise came in handy... For this board, Meg and I went with Milk Duds, Sour Patch Kids, Red Vines, Junior Mints, Sour Straws, Swedish Fish, yogurt-covered pretzels, Bunch of Crunch, popcorn, M&Ms, Good & Plenty, Hot Tamales, Mike & Ikes, and Whoppers, for a perfect mix of gummy and chocolate. 2.Start with ""grounding points." "Similar to placing cheese on a cheese board, you want to start with ""grounding points,"" which, in this case, are ramekins filled with candy."
