# Supplementary figures and images for: Differences in fractal patterns and characteristic periodicities between word salads and normal sentences: Interference of meaning and sound
Source: PLoS One. 2021 Feb 18;16(2):e0247133. doi: 10.1371/journal.pone.0247133 (PMC7891721; doi:10.1371/journal.pone.0247133)

## Slide 1
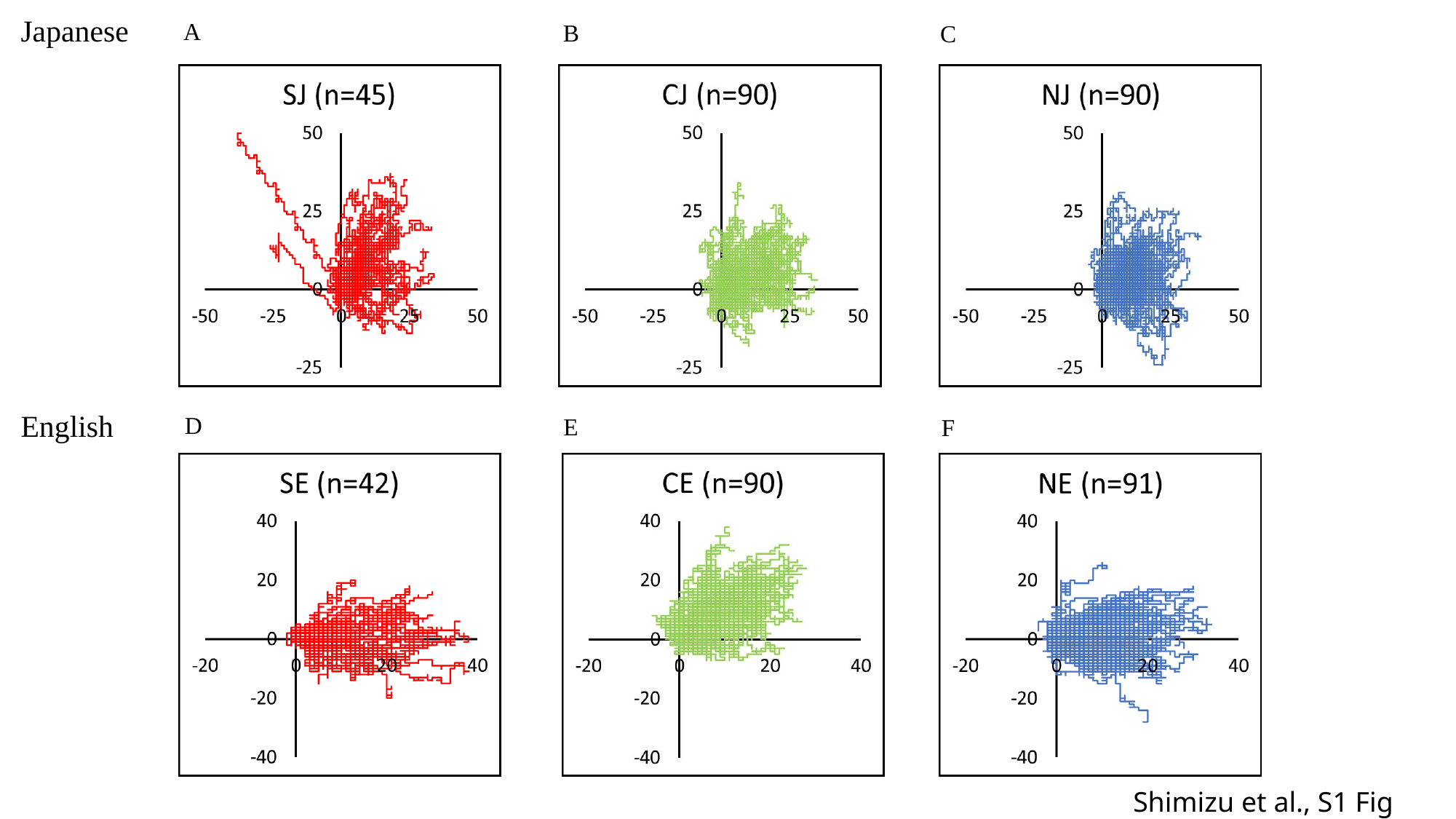

Japanese
A
B
C
English
D
E
F
Shimizu et al., S1 Fig

Supplement: S1 Fig — (A) Random walks in Japanese analyzed for (A) normal sentences (NJ) are plotted in blue. (B) Random walks for computer-generated word salads (CJ) are plotted in green. (C) Random walks for word salads of schizophrenia patients (SJ) plotted in red. Random walks in English analyzed for (D) normal sentences (NE) are plotted in blue. (E) Random walks for computer generated word salad (CE) are plotted in green. (F) Random walks for word salads of schizophrenia patients (SE) are plotted in red. (PPTX) [file pone.0247133.s002.pptx]

## Slide 1
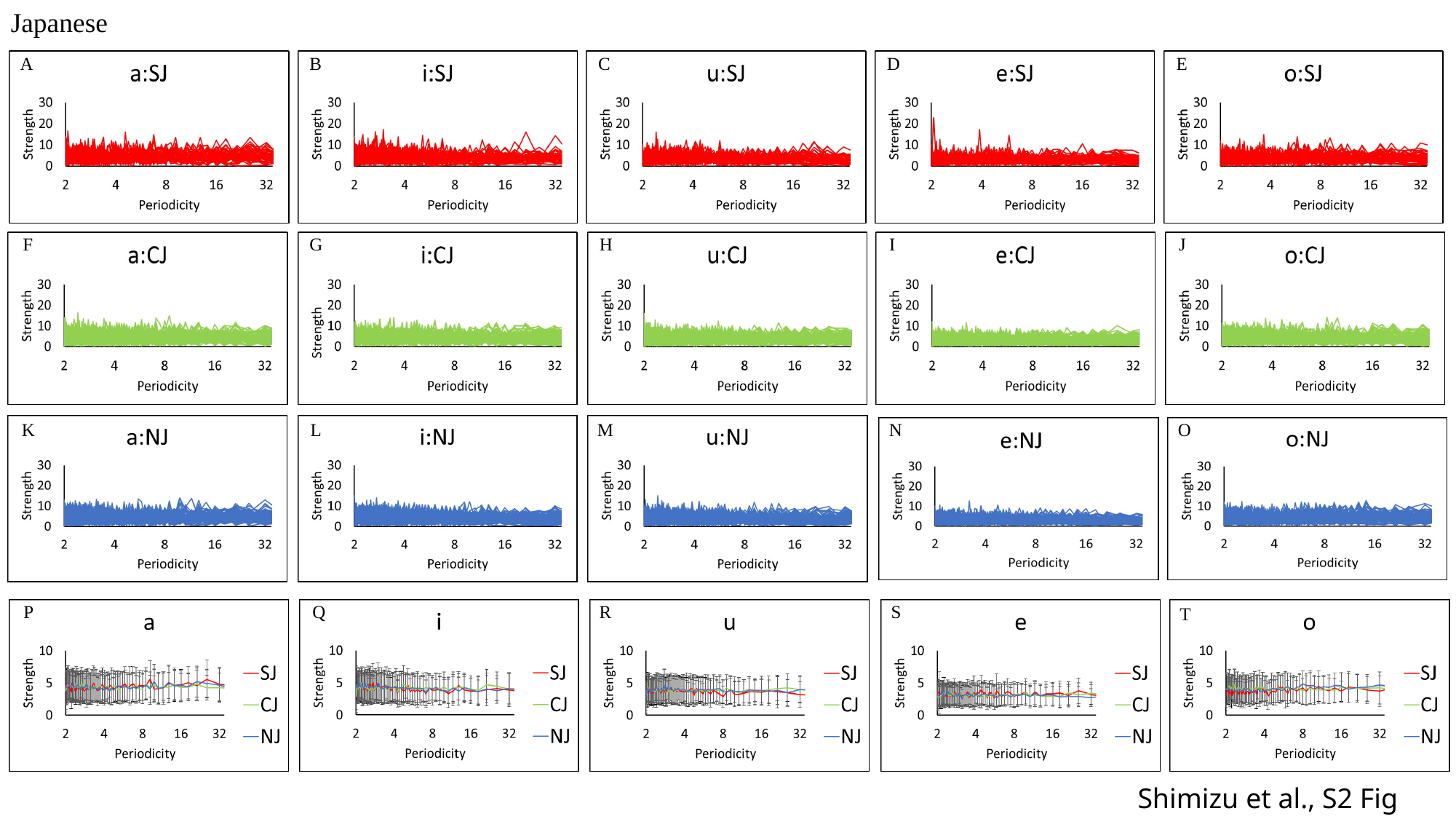

Japanese
A
B
C
D
E
F
G
H
I
J
K
L
M
N
O
P
Q
R
S
T
Shimizu et al., S2 Fig

Supplement: S2 Fig — Power as a function of periodicity for random walk of all the data in Japanese: NJ (A–E), CJ (F–J), and SJ (K–O). (P–T) Mean and standard deviation of power at each integer periodicity are shown for NJ (blue), CJ (green), and SJ (red). (PPTX) [file pone.0247133.s003.pptx]

## Slide 1
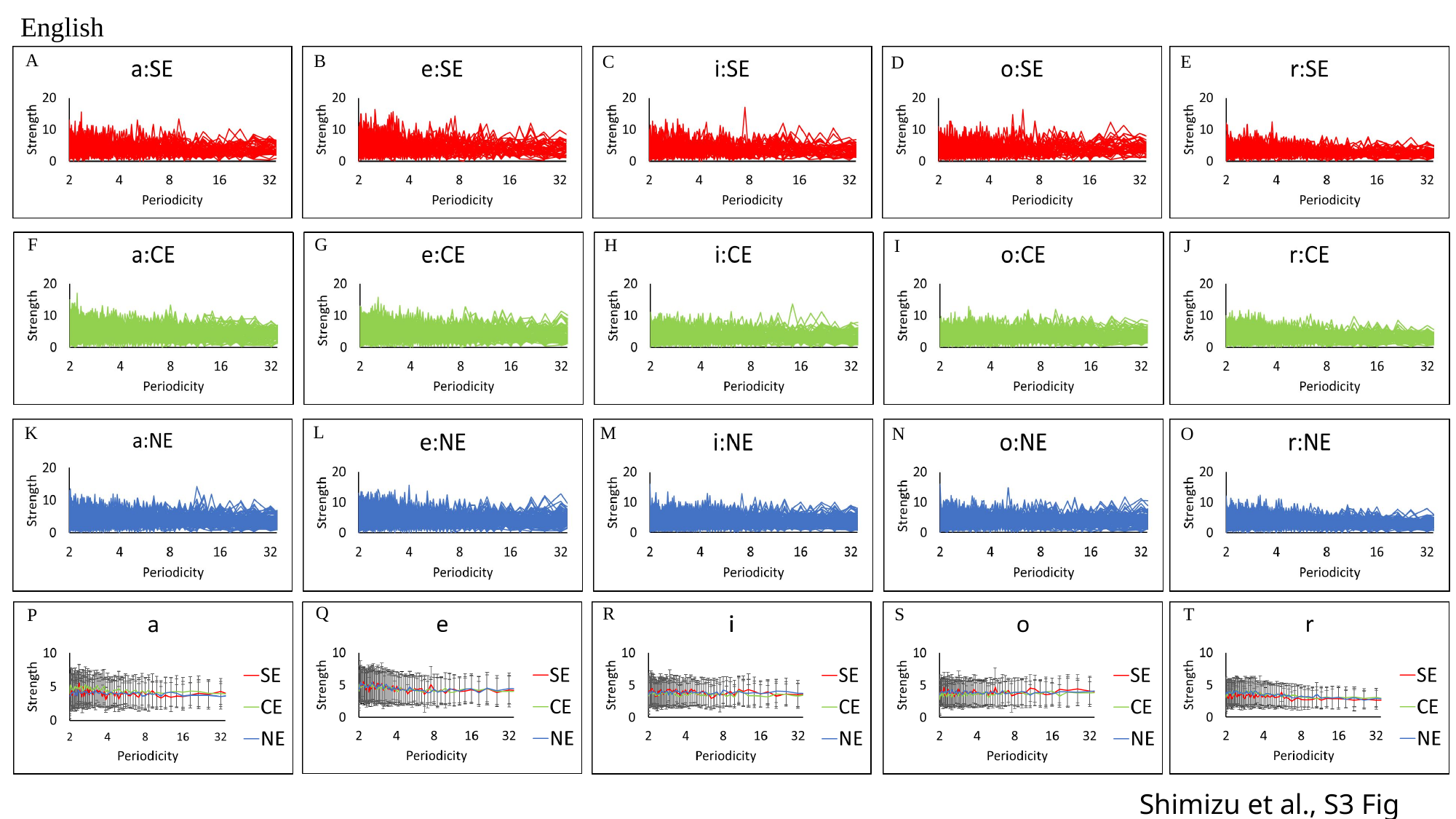

English
A
B
E
C
D
F
G
H
I
J
L
M
K
N
O
Q
R
S
T
P
Shimizu et al., S3 Fig

Supplement: S3 Fig — Power as a function of periodicity for random walk of all the data in English: NE (A–E), CE (F–J), and SE (K–O). (P–T) Mean and standard deviation of power at each integer periodicity are shown for NE (blue), CE (green), and SE (red). (PPTX) [file pone.0247133.s004.pptx]

## Slide 1
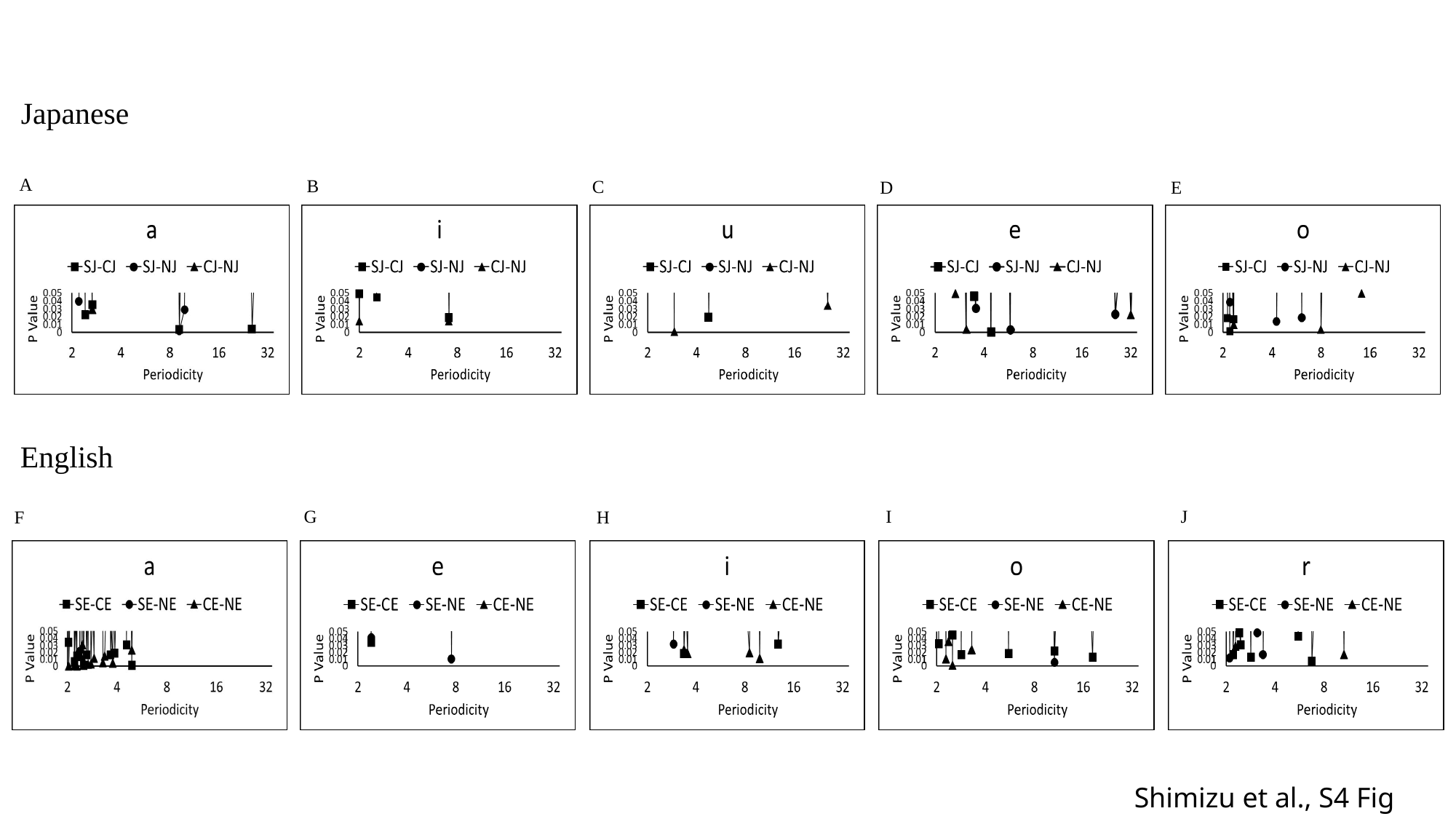

Japanese
A
B
C
D
E
English
G
I
J
H
F
Shimizu et al., S4 Fig

Supplement: S4 Fig — (A–E) P-values for the difference between SJ and CJ, SJ and NJ, and CJ and NJ are plotted as a function of periodicity. (F–J) P-values for the difference between SE and CE, SE and NE, and CE and NE are plotted as a function of periodicity. (PPTX) [file pone.0247133.s005.pptx]
