# Supplementary material for: Differences in fractal patterns and characteristic periodicities between word salads and normal sentences: Interference of meaning and sound
Source: PLoS One. 2021 Feb 18;16(2):e0247133. doi: 10.1371/journal.pone.0247133 (PMC7891721; doi:10.1371/journal.pone.0247133)
Supplement: S1 Table — (DOCX) [file pone.0247133.s006.docx]

S1 Table. Summary of patients information in SJ.

－: unknown

| Data No. | Diagnosis | DSM-5 | Age | Gender | main symptoms |
| --- | --- | --- | --- | --- | --- |
| 1 | Schizophrenia | Ⅱ | 40 | F | word salad, delusion, hallucination |
| 2 | Schizophrenia | Ⅱ | － | － | word salad, |
| 3 | Schizophrenia | Ⅱ | 58 | M | word salad, disturbance, |
| 4 | Schizophrenia | Ⅱ | 25 | F | word salad, delusion |
| 5 | Wernicke's encephalopathy | Ⅱ | 63 | M | word salad, neologism, disturbance, |
| 6 | Schizophrenia | Ⅱ | 25 | F | word salad, delusion, hallucination |
| 7 | Schizophrenia | Ⅱ | 39 | M | word salad, delusion |
| 8 | Schizophrenia | Ⅱ | 15～45  (17 person） | F  M | word salad,  hallucination |
| 9 |  |  |  |  |  |
| 10 |  |  |  |  |  |
| 11 |  |  |  |  |  |
| 12 | Schizophrenia | Ⅱ | 19 | M | word salad, neologism, disturbance |
| 13 | Schizophrenia | Ⅱ | 43 | F | word salad, delusion, hallucination |
| 14 | Schizophrenia | Ⅱ | 60 | M | word salad, disturbance, delusion |
| 15 | Delusional Disorder | Ⅱ | 72 | F | word salad, delusion |
| 16 | Schizophrenia | Ⅱ | 18 | F | word salad, delusion, hallucination |
| 17 | Schizophrenia | Ⅱ | － | － | word salad, disturbance, delusion |
| 18 |  |  |  |  |  |
| 19 | Schizophrenia | Ⅱ | 48 | F | word salad, disturbance |
| 20 |  |  |  |  |  |
| 21 | Schizophrenia | Ⅱ | 42 | F | word salad, delusion, hallucination |
| 22 | Senile psychosis | Ⅱ  XVII | 65 | F | word salad, delusion, hallucination |
| 23 | Schizophrenia | Ⅱ | － | － | word salad, neologism, disturbance, |
| 24 | Schizophrenia | Ⅱ | 23 | F | word salad, delusion |
| 25 | Schizophrenia | Ⅱ | － | F | word salad, disturbance |
| 26 | Tuberous  sclerosis | Ⅱ | 43 | M | word salad, neologism, disturbance, |
| 27 | Schizophrenia | Ⅱ | － | － | word salad |
| 28 | Schizophrenia | Ⅱ | 39 | M | word salad, delusion, hallucination |
| 29 | Schizophrenia | Ⅱ | 40 | M | word salad, disturbance, |
| 30 | Schizophrenia | Ⅱ | － | － | word salad,  neologism,  disturbance,  delusion |
| 31 |  |  |  |  |  |
| 32 |  |  |  |  |  |
| 33 |  |  |  |  |  |
| 34 | Schizophrenia | Ⅱ | 55 | M | word salad, delusion, hallucination |
| 35 | Schizophrenia | Ⅱ | 58 | M | word salad, neologism |
| 36 | Schizophrenia | Ⅱ | 35 | M | word salad, delusion, hallucination |
| 37 | Schizophrenia | Ⅱ | 20 | F | word salad, delusion, hallucination |
| 38 | Schizophrenia | Ⅱ | 21 | M | word salad, delusion, hallucination |
| 39 | Schizophrenia | Ⅱ | 30 | M | word salad, delusion, hallucination |
| 40 | Schizophrenia | Ⅱ | 51 | F | word salad, delusion, hallucination |
| 41 | Schizophrenia  Pervasive, developmental disorders | Ⅱ  Ⅰ | － | － | word salad,  neologism,  disturbance,  delusion |
| 42 | Schizophrenia | Ⅱ | 44 | M | word salad, neologism |
| 43 | Schizophrenia | Ⅱ | 60 | F | word salad, delusion |
| 44 | Schizophrenia | Ⅱ | 52 | F | word salad, delusion, disturbance, |
| 45 | Schizophrenia | Ⅱ | 55 | M | word salad, delusion, hallucination |
