# Supplementary material for: Differences in fractal patterns and characteristic periodicities between word salads and normal sentences: Interference of meaning and sound
Source: PLoS One. 2021 Feb 18;16(2):e0247133. doi: 10.1371/journal.pone.0247133 (PMC7891721; doi:10.1371/journal.pone.0247133)
Supplement: S2 Table — (DOCX) [file pone.0247133.s007.docx]

S2 Table. Summary of patients in formation in SE

－: unknown

| data No. | Diagnosis | DSM-5 | Age | Gender | main symptoms |
| --- | --- | --- | --- | --- | --- |
| 1 | Schizophrenia,  Bipolar Disorders,  manic state,  Depression | Ⅱ  Ⅲ | － | － | word salad,  flight of ideas,  delusion,  hallucination,  disturbance, |
| 2 |  |  |  |  |  |
| 3 |  |  |  |  |  |
| 4 |  |  |  |  |  |
| 5 |  |  |  |  |  |
| 6 |  |  |  |  |  |
| 7 |  |  |  |  |  |
| 8 |  |  |  |  |  |
| 9 |  |  |  |  |  |
| 10 |  |  |  |  |  |
| 11 |  |  |  |  |  |
| 12 |  |  |  |  |  |
| 13 |  |  |  |  |  |
| 14 | Schizophrenia,  Dementia | Ⅱ  XVII | － | － | word salad |
| 15 |  |  |  |  |  |
| 16 |  |  |  |  |  |
| 17 |  |  |  |  |  |
| 18 | Schizophrenia | Ⅱ | － | － | word salad, delusion |
| 19 |  |  |  |  |  |
| 20 | Schizoaffective Disorder | Ⅱ | － | － | word salad, delusion |
| 21 | Schizophrenia  Drug-related toxic psychosis | Ⅱ  XVI | 16～39  （17 person） | F  M | word salad,  delusion,  hallucination |
| 22 |  |  |  |  |  |
| 23 |  |  |  |  |  |
| 24 |  |  |  |  |  |
| 25 | Schizophrenia, Paranoid | II  XVIII | － | － | word salad, disturbance |
| 26 | Risk Mental State (ARMS) | Ⅱ | 17～20  （5 person） |  | word salad,  disturbance |
| 27 |  |  |  |  |  |
| 28 | Schizophrenia | Ⅱ | － | － | word salad, disturbance, |
| 29 | Schizophrenia  Schizoaffective Disorder | Ⅱ | －  （15 person） | F  M | word salad,  delusion,  hallucination |
| 30 | Schizophrenia | Ⅱ | － | － | word salad, disturbance, delusion |
| 31 | Schizophrenia | Ⅱ | － | － | word salad, disturbance, |
| 32 | Schizophrenia  Drug-related toxic psychosis | Ⅱ  XVI | 18～43  （4 person） | F  M | word salad, delusion, |
| 33 | Autism | Ⅰ  Ⅱ | －  （5 person） | F  M | word salad, |
| 34 | Schizophrenia | Ⅱ | 18～25  （3 person） | M | word salad, delusion, |
| 35 | Paraphrenia | Ⅱ | 63 | F | word salad, delusion, |
| 36 | Schizophrenia | Ⅱ | 25 | M | word salad, delusion, |
| 37 | Schizophrenia | Ⅱ | － | － | word salad,  delusion,  hallucination, |
| 38 |  |  |  |  |  |
| 39 |  |  |  |  |  |
| 40 | Schizophrenia | Ⅱ | 24～62  （11 person） | F  M | word salad, delusion, |
| 41 | Schizophrenia | Ⅱ | 15～50  （5 person） | F  M | word salad, hallucination, delusion |
| 42 | Cenesthopathy | Ⅱ | 16～26  （10 person） | F  M | word salad, hallucination, delusion |
